# Cataract Aggravates Alzheimer-Like Pathologies and Cognitive Deficits in an APP/PS1 Mouse Model

**DOI:** 10.1007/s12264-025-01442-z

**Published:** 2025-06-28

**Authors:** Zhao Geng, Zhong-Yuan Yu, Jun Tan, Xuan-Yue Wang, Gui-Hua Zeng, Jiang-Hui Li, Yu-Di Bai, Xiao-Qin Zeng, Yu-Peng Zhu, Cheng-Rong Tan, An-Yu Shi, Yu-Hui Liu, Xian-Le Bu, Zi Ye, Yan-Jiang Wang, Zhao-Hui Li

**Affiliations:** 1 Senior Department of Ophthalmology, The Third Medical Centre of PLA General Hospital, Beijing, 100853 China; 2 Department of Neurology and Centre for Clinical Neuroscience, Daping Hospital, Third Military Medical University, Chongqing, 400042 China; 3 Institute of Brain and Intelligence, Third Military Medical University, Chongqing, 400038 China; 4 Chongqing Key Laboratory of Aging and Brain Diseases, Chongqing, 400042 China; 5 Department of Neurology, The First Affiliated Hospital of Wenzhou Medical University, Wenzhou, 325000 China; 6 Department of Ophthalmology, Daping Hospital, Third Military Medical University, Chongqing, 400042 China

**Keywords:** Cataract, Alzheimer’s disease, Aβ burden, Visual impairment, Behavioural deficits

## Abstract

Clinical investigations have suggested a potential link between cataracts and Alzheimer’s disease (AD). However, whether cataract has an impact on the progression of AD remains unclear. The objective of this research was to determine the relationship between cataracts and AD. A cataract model was established in APP/PS1 [mutant amyloid precursor protein (APP) and a mutant presenilin-1 (PS1) gene] mice *via* lens puncture. Behavioural assays were used to evaluate cognitive function. Immunohistochemistry, immunofluorescence, and enzyme-linked immunosorbent assays (ELISA) were applied to detect AD-related pathology. Visual signals were markedly obstructed following surgery to induce cataracts, and these mice presented an increased cerebral amyloid-beta (Aβ) load, while no significant alterations in the levels of enzymes associated with Aβ metabolism were detected. In addition, compared with control mice, cataract model mice presented increased astrogliosis and microgliosis, along with elevated levels of proinflammatory factors. Moreover, cataract model mice presented more pronounced cognitive impairments than did control mice. Our study offers experimental confirmation that cataract considerably contributes to the pathogenesis of AD, thereby emphasizing the importance of visual signals in maintaining cognitive well-being.

## Introduction

The brain serves as the central hub for perception and understanding of the world, but cognitive processes do not occur in isolation. The brain also guides our behaviour by receiving information from the external environment through the visual, auditory, tactile, olfactory, and gustatory systems, and guides our behaviour accordingly [1]. Among these, 80% to 90% of external information is derived from vision, which primarily builds cognitive, memory, and learning abilities through recognition and perception [1]. Therefore, visual signals play an important role in the human brain’s process of perceiving the outside world and constructing cognition.

In recent years, numerous studies have indicated that visual impairment (VI) is a risk factor for cognitive impairment [2–4]. Alzheimer’s disease (AD) is the most common type of dementia, accounting for ~60%–80% of cases of cognitive impairment, and is characterized by progressive memory loss and cognitive deficits [5]. Approximately 44 million individuals worldwide are afflicted with AD, and the global prevalence is estimated to increase to 130 million by 2050 [6]. Cataract, as the leading cause of VI worldwide, accounts for ~46% of the global cases of blindness [7]. Epidemiological studies have found that cataracts and AD share some common risk factors, such as aging, female gender, obesity, smoking, and vascular abnormalities. Moreover, AD patients show a high prevalence of comorbid cataracts [8]. Previous research has also revealed that Aβ in the lens can promote the aggregation of lens proteins, thereby affecting light transmittance. Our group has previously reported elevated levels of Aβ in the aqueous humour of eyes in patients with age-related cataracts [9, 10]. This suggests that cataracts may play a role in the development of AD. However, to our knowledge, experimental evidence establishing a causal relationship between AD and cataracts remains scarce.

To elucidate the relationship between cataracts and the development of AD, we constructed a cataract model with the APP/PS1 [mutant amyloid precursor protein (APP) and a mutant presenilin-1 (PS1) gene] mouse and examined the influence of cataracts on AD-like pathologies and cognitive functions. Our findings demonstrate that cataract significantly contributes to the pathogenesis of AD.

## Materials and Methods

### Mice

Female APPswe/PS1dE9 transgenic mice on the C57BL/6 background, which were genetically modified to harbour AD-linked mutations, namely, chimeric mouse/human APP695 with the Swedish mutation (K595N/M596L) and human PS1 lacking exon 9 (PS1dE9), both under the control of the prion promoter [11, 12], were obtained from the Jackson Laboratory (Bar Harbor, ME, USA; JAX catalogue number: 005864). Female wild-type C57BL/6J mice were provided by the Animal Centre of Daping Hospital affiliated with the Third Military Medical University. The ethical and welfare aspects of all the experimental protocols were approved by the Laboratory Animal Welfare and Ethics Committee of Daping Hospital (AMUWEC20237384).

### Establishment of the Cataract Model

Cataracts were induced in 6-month-old AD model mice. The eyes of the mice were dilated with tropicamide eye drops (Santen, Osaka, Japan). Under the maintenance inhalation anaesthesia with 1.5% isoflurane (Ruiwode Life Science, Shenzhen, China), the eyelids were disinfected with iodine. Under a microscope, a 30G needle was inserted from the lateral side of the limbus cornea to puncture the lens. After the procedure, levofloxacin eye drops were applied to both eyes to prevent infection (Santen, Osaka, Japan). The mice were then placed on a warm pad for recovery. The control group was anaesthetized and underwent the same procedure except for cataract induction. The clinical symptoms of the cataract model mice were examined *via* a slit lamp.

### Light‒Dark Box Test

At 12 months of age, all the experimental mice were transferred from their cages to a behavioural testing room for at least 30 min for acclimation to the new environment. We prepared a 30 cm × 60 cm × 30 cm box and divided it into two compartments of equal size, with a 5 cm diameter opening in the middle to allow the mice to pass through. Then, we placed a mouse in the centre of the illuminated compartment with its back to the partition, activated the camera and lighting equipment, and began recording the mouse’s behaviour. After a 5-min recording session, the mouse was removed from the experimental box and placed in its cage. The videos were analysed *via* EthoVision XT behavioural tracking software (Noldus Information Technology Inc., Wageningen, Netherlands).

### Visual Evoked Potential (VEP) Recording

Each mouse was appropriately anaesthetized, treated with a mydriatic agent, and then placed on a heating pad to maintain a constant body temperature. The skin was cleaned, and electrodes were placed to ensure good contact with the skin. Scalp electrodes were positioned over the corresponding bony landmarks, subcutaneously at the inion along the line connecting both ears, and at the midpoint of the line connecting both eyes. The ground wire was connected to the tail. A full-field flash stimulator was used (Retiminer-S, Chongqing, China). After stimulation, the animals were removed from the stimulation environment and allowed to recover on a heating pad. RetiMINER 3.0 was applied to analyse the VEP waveforms and corresponding parameters, such as latency.

### Behavioural Tests

At 12 months of age, all the experimental mice were subjected to the Y-maze novel arm test, the open field test, and the fear conditioning test. All the mice were placed in an experimental room to become familiar with the environment before the formal experiments. The Y-maze novel arm test was conducted to assess spatial learning and memory. One arm of the Y-maze was blocked, representing the novel arm. Each mouse was placed in the starting arm and allowed to move freely in the starting arm and the other arm for a certain period (usually 300 s). After the training session, the mouse was returned to its cage to rest for a period (usually 1 h). During the test phase, the barrier blocking the novel arm was removed, allowing a mouse to explore all three arms freely. Each mouse was placed in the starting arm again, and the number of entries into and time spent in the new arm, the starting arm, and the other arm were recorded. Behaviour was tracked with a computer tracking system (ANY-maze, Stoelting, Wood Dale, USA). The open field test was utilized to assess locomotor activity. After acclimatization, each mouse was gently placed in the centre of the open field chamber with its back to the experimenter, who then left the area. The activity of the animals in the open field chamber was recorded for > 5 min (ANY-maze, Stoelting). Behavioural observations included dynamic tracking, total distance travelled, and velocity. The fear conditioning test was applied to assess memory. Initially, each mouse was placed in a 20 cm × 20 cm × 20 cm square chamber and allowed to adapt for 2 min. Then, it was presented with three auditory conditioned stimuli (CSs; 80 dB at 4 kHz for 30 s) paired with a foot shock (unconditioned stimulus; 0.3 mA for 2 s) within 28 s after the onset of the CS at 60 s intervals. After the training session, the experimental chamber was cleaned. Twenty-four hours later, each mouse was placed back in the chamber and presented with three auditory CSs to test auditory-cued fear memory. Their freezing behaviour was recorded for 5 min and analysed using EthoVision XT behavioural tracking software (Noldus Information Technology Inc.).

### Brain Sampling

All the mice were humanely sacrificed, and brain samples were obtained in accordance with previously established methods [13]. After being anaesthetized, each mouse was intracardially perfused with a solution of 0.1% NaNO_2_ in normal saline. The right cerebral hemisphere was then dissected, fixed in 4% paraformaldehyde, and 30-μm coronal sections were cut on a freezing microtome (Leica, Weztlar, Germany) for subsequent immunofluorescence or immunohistochemical staining. The left cerebral hemisphere was rapidly frozen in liquid nitrogen, pulverized into a powder, portioned into three vials, weighed, and preserved at −80 °C for subsequent biochemical analysis.

### Detection of Aβ Plaques

Five representative sections spanning the entire brain were stained with a 6E10 antibody (1:1000, Biolegend, San Diego, USA) or thioflavin S (0.015%, Sigma, Saint Louis, USA) to identify Aβ plaques. The methods used were described in our previous study [13, 14]. All the sections were photographed with an Olympus VS200 slide scanner (Olympus, Tokyo, Japan) or Zeiss microscope (Zeiss, Oberkochen, Germany), and the images were analysed with ImageJ software under the same conditions by investigators who were blinded to the group information.

### Investigation of Microglial Uptake of Aβ

Two representative sections spanning the brain regions containing the visual cortex were selected. After being washed and permeabilized with 0.5% Triton-100X (Sangon, Shanghai, China) and blocked with 3% bovine serum albumin (BSA, Sigma), the sections were incubated overnight with 6E10 (1:1000, Biolegend) and anti-IBA1 (1:1000, Wako, Tokyo, Japan) primary antibodies. The following day, the sections were incubated with secondary antibodies conjugated to different fluorophores, and then images were captured using a confocal microscope (Zeiss). The colocalization of the fluorescence signals was analysed using ImageJ software (National Institutes of Health, Bethesda, USA).

### Western Blotting

Brain samples were homogenized in ice‐cold RIPA lysis buffer (Keygentec, Nanjing, China). Proteins were separated *via* 4%–20% SDS-polyacrylamide gel electrophoresis (Genscript, Nanjing, China). The separated proteins were electrotransferred onto nitrocellulose membranes (Millipore, Bedford, USA). The membranes were blocked with 3% BSA and incubated with primary antibodies overnight at 4 °C. The primary antibodies used were: anti‐APP C‐terminal (1:1000, Millipore), anti‐BACE1 (1:1000, Abcam, Cambridge, UK), anti‐ADAM10 (1:1000, Abcam), anti‐PS1 (1:1000, Millipore), anti‐IDE (1:1000, Millipore, Bedford, USA), anti‐RAGE (1:1000, Millipore), anti‐LRP1 (1:1000, Abcam), and anti‐β‐actin (1:2000, Sigma). The membrane was washed and incubated with the corresponding secondary antibodies, and then scanned on an Odyssey fluorescent scanner (Odyssey, Massachusetts, USA). The intensity of each band was normalized to the intensity of the internal reference protein band for analysis.

### Investigation of Neuroinflammation

Astrocytes were labelled with an anti-GFAP antibody (1:1000, Abcam). Activated microglia were labelled with an anti-CD68 antibody (1:200, Abcam). All histological staining was performed as described in our previous study [13, 14]. All the sections were photographed with an Olympus VS200 slide scanner, and the images were analysed with ImageJ software under the same conditions by investigators blinded to the group information.

### ELISA

Proteins were extracted from brain tissue samples using RIPA lysis buffer. The levels of the inflammatory factors TNF‐α, IL‐1β, and IL‐6 in brain homogenates were measured with ELISA kits (Raybiotech, Peachtree Corners, USA) and normalized to the total protein levels.

### Statistical Analysis

All the data are presented as the mean ± SEM and were analysed with SPSS 20.0 software (IBM, Chicago, USA). The statistical figures were produced by GraphPad Prism (version 10.1.2, GraphPad Software, San Diego, USA). Unpaired *t*-tests and Mann–Whitney *U* tests were used to assess the significance of differences between the two groups. The selection of the unpaired *t*-test or Mann–Whitney *U* test depended on the normality of the data. The differences were considered statistically significant at *P* <0.05.

### Data and Material Availability

The datasets generated during the current study are not publicly available, but are available from the corresponding author on reasonable request.

## Results

### Cataract Causes Visual Impairment in AD Model Mice

**Fig. 1:**
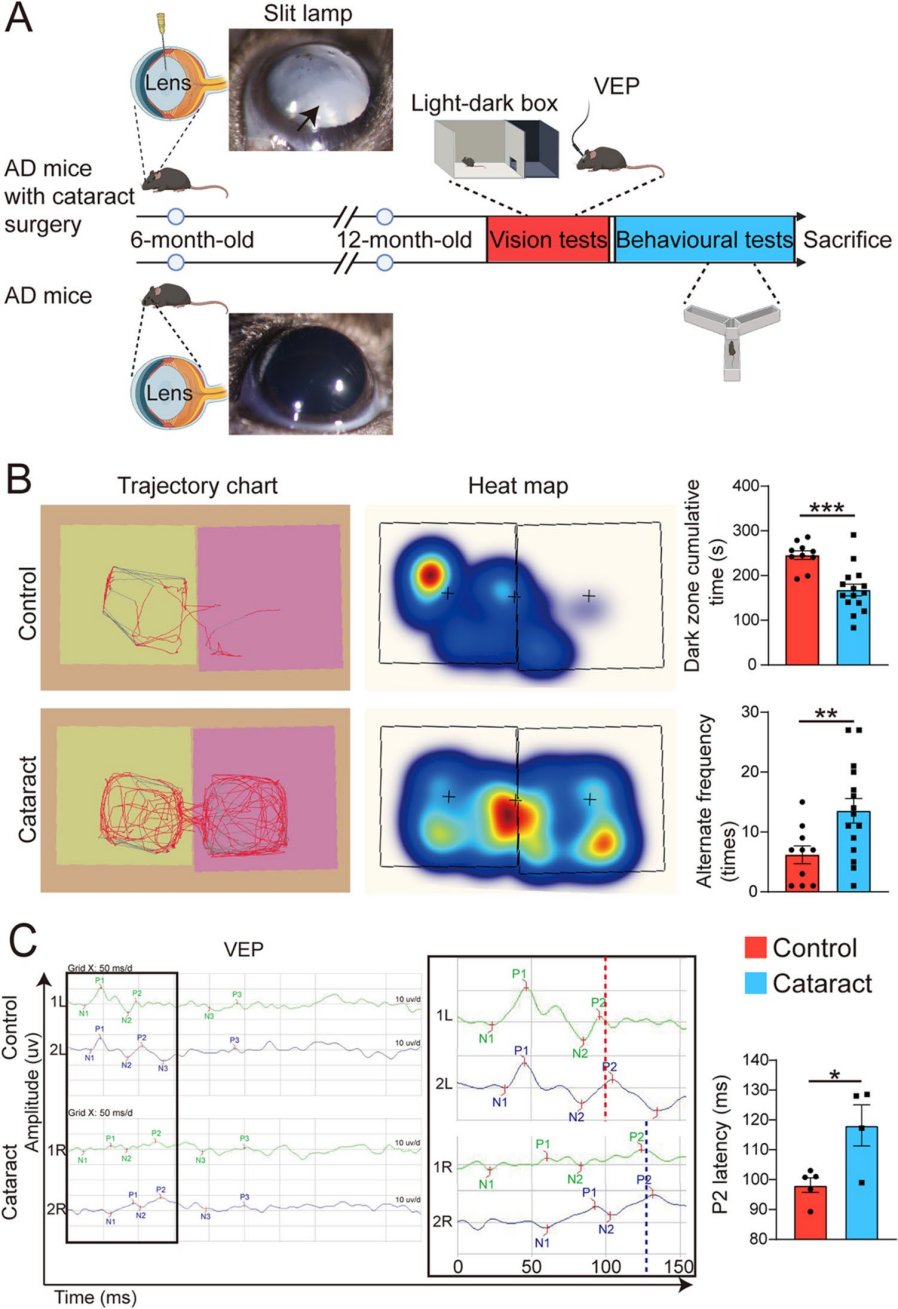
Establishment of the cataract model and visual acuity assessment. **A** Schematic of the basic experimental procedure of this study. Representative anterior segment slit lamp photographs of mice in the control group and the cataract group are displayed in the flowchart. The arrow points to an opacified lens. **B** Representative data from the light‒dark box test and statistical analysis of the data. The trajectory maps show the activity of the mice during the test, and the red line represents a strong recorded signal, while the gray line indicates a weaker signal. The heatmaps show the distribution of activity throughout the box during the test, and the shift from cold colors (blue) to warm colors (red) indicates a gradual increase in the activity level at that location. The cumulative time in the dark zone was analyzed using an unpaired *t*-test, while the alternate frequency was assessed using Mann–Whitney *U* tests. *n* = 10–15. **C** Representative images of VEPs and statistical analysis (unpaired *t*-tests) of the data. In the *y*-axis, R represents the right eye, and L represents the left eye; The numbers indicate the waveforms from the different detections. N1 indicates the occurrence of the first negative wave, and N2 indicates the second negative wave. P1 represents the first positive wave, and P2 represents the second positive wave. The black box on the right is an enlarged image of the black box on the left. *n* = 4–5. *P <0.05, **P <0.01, and ***P <0.001. The error bars are the SEMs.

First, we established a cataract model as shown in the flowchart depicted in Fig. 1A. The cataract model was generated with APP/PS1 mice (also referred to as AD mice) at age 6 months, at which point AD pathology had begun to develop. On the 7th day after modelling, the slit-lamp examination was conducted to confirm the successful establishment of the cataract model (Fig. 1A). The lenses of the mice in the AD-cataract group (hereafter referred to as the cataract group) were obviously opaque. At 12 months of age, to assess the visual acuity of the mice, we conducted a light‒dark box test. In this test, mice with normal vision typically exhibit photophobia, thus spending more time exploring the dark area and exhibiting a reduced number of transitions between the light and dark compartments. The results revealed a significant decrease in the total time spent in the dark area and an increased number of light‒dark transitions in the cataract group compared with the control group (Fig. 1B), indicating visual impairment in the cataract mice. Given that anxiety may affect the outcomes of the light‒dark box test, to avoid confounding factors, we also recorded the visual evoked potential (VEP) to assess visual impairment from a physiological perspective. Consistently, we found significant prolongation of the P2 wave latency in the cataract group compared with the control group, confirming a decline in visual acuity (Fig. 1C). Our results suggest that cataract causes visual impairment in AD model mice.

### Cataract Exacerbates the Cognitive Deficits in AD Model Mice

**Fig. 2:**
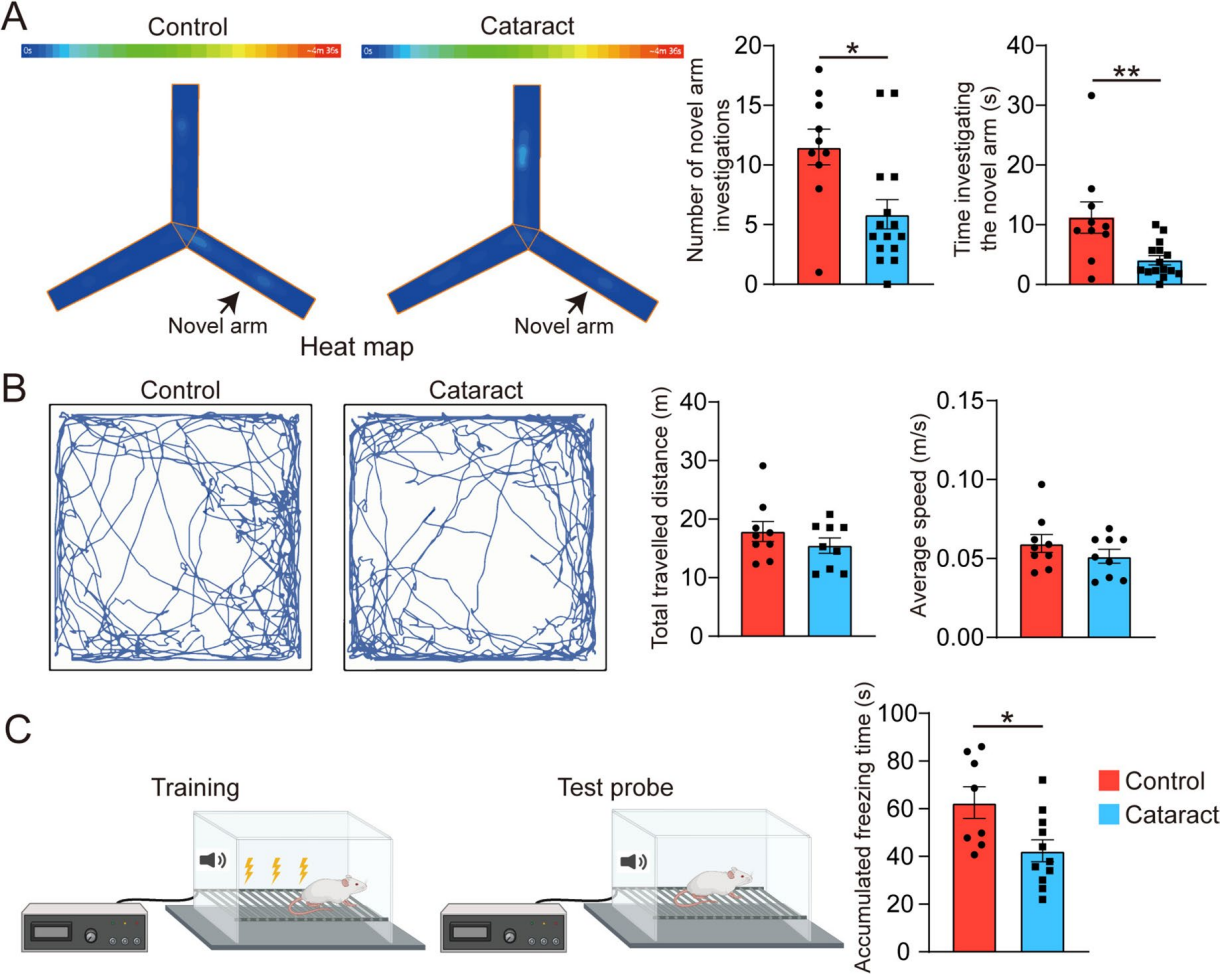
Assessment of the impact of cataracts on the behaviour of AD model mice. **A** Representative heatmaps of the two groups of mice in the Y-maze novel arm test and statistical analysis of the data (Mann–Whitney *U* tests). The color bar at the top indicates the activity levels in the heatmap below. The warmer tones towards the right side indicate higher activity in those regions. *n* = 10–14. **B** Representative trajectories of the two groups of mice in the open field test and statistical analysis of the data (unpaired *t*-tests). *n* = 9. **C** Schematic of the experimental procedure of the fear conditioning test and statistical analysis of the data (unpaired *t*-tests). *n* = 8–11. *P <0.05, **P <0.01. The error bars are the SEMs.

Subsequently, at 12 months of age, we evaluated the cognitive function of cataract model mice *via* the Y-maze, open field, and fear conditioning tests. The heatmaps of the mice in the Y maze test are depicted in Fig. 2A. Compared with the cataract group, the control group exhibited a greater number of entries into the novel arm and a longer exploration time in the novel arm, indicating an increased tendency for spatial exploration and better short-term memory. In addition, we conducted an open field test to assess the locomotor ability of the mice. Fig. 2B displays the trajectories of the mice in both groups. Although there were no significant differences between the two groups in the total distance travelled or average speed, a trend towards a decrease in both of these parameters was found in the cataract group. Furthermore, to mitigate the influence of impaired vision on cognitive function as much as possible, we applied the fear conditioning test, which does not require vision. The cumulative freezing time in the cataract group was shortened, indicating poorer cognitive function in the cataract group than in the control group (Fig. 2C). In summary, consistent with our previous studies, these behavioural results show that cataracts significantly exacerbate cognitive deficits in AD model mice.

### Cataract Exacerbate Aβ Deposition in the Brain

**Fig. 3:**
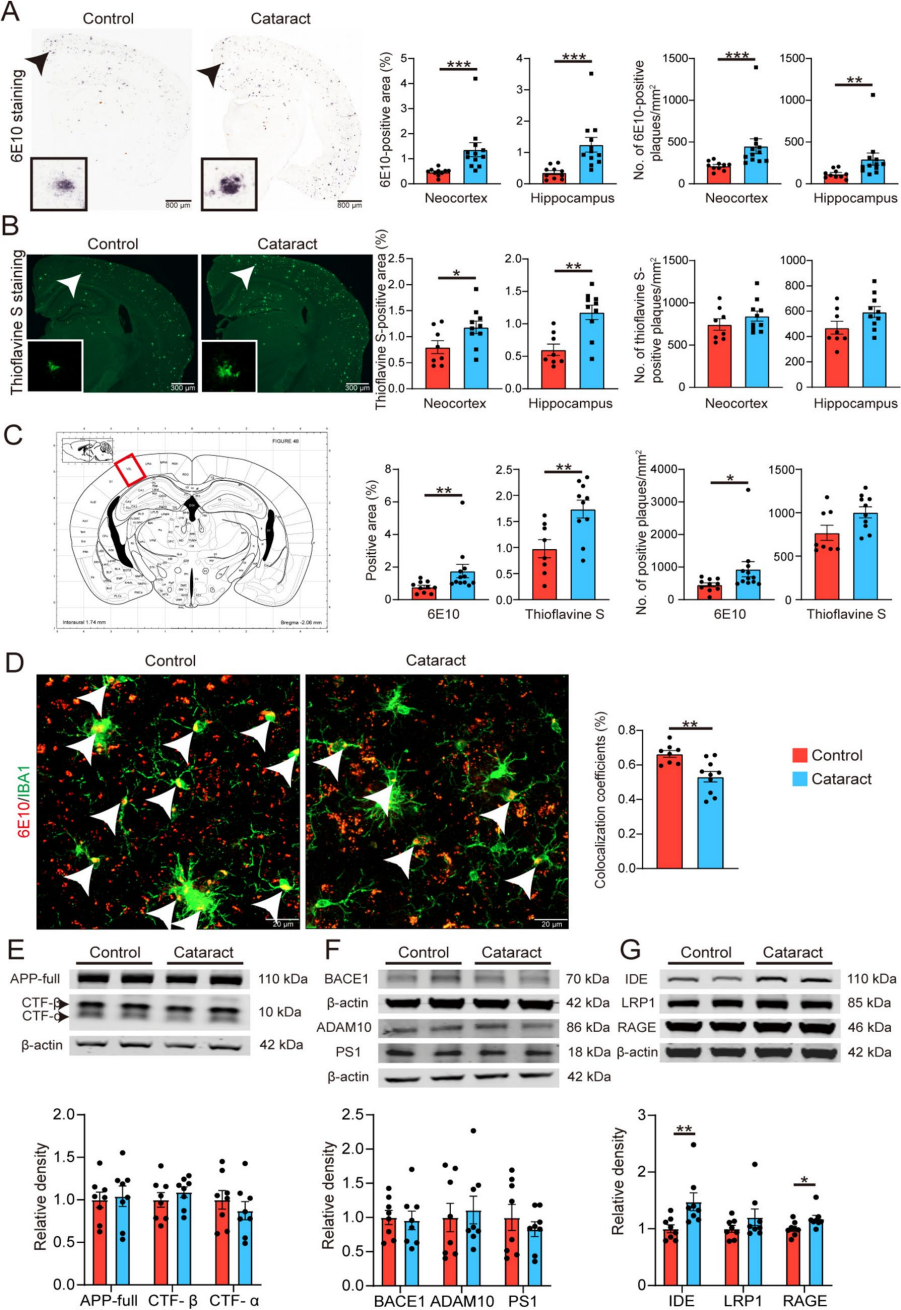
Cataract exacerbates the Aβ plaque burden in the brains of AD model mice. **A** Representative images of staining with the 6E10 antibody in brain sections from cataract and control groups and quantitative results for the neocortex and hippocampus (Mann–Whitney *U* tests). Scale bars, 800 µm. Insets: magnified views of the areas indicated by the arrows. *n* = 10–12. **B** Representative images of thioflavin S staining in brain sections from cataract and control group mice and quantitative results for the neocortex and hippocampus (unpaired *t*-tests). Scale bars, 800 µm. Insets: magnified view of the areas indicated by the arrows. *n* = 8–10. **C** Left, image of a coronal brain section; red square indicates the visual cortex. Right, statistical analysis of the numbers of 6E10- and thioflavin S-positive plaques in the visual cortex (the statistical charts from left to right represent three consecutive unpaired *t*-tests followed by one Mann–Whitney *U* tests). **D** Representative images of 6E10 and IBA1 co-staining in the visual cortex of cataract and control group mice and quantitative results (unpaired *t*-tests). Scale bars, 20 µm. The white arrow indicates the co-staining area, which represents the region where microglia phagocytize Aβ. **E** Representative Western blots and quantitative analysis of the levels of APP and its metabolites (CTF-β, CTF-α) in brain homogenates (unpaired *t*-tests). **F** Representative Western blots and quantitative analysis of APP‐metabolizing enzyme levels in brain homogenates (unpaired *t*-tests). **G** Representative Western blots and quantitative analysis of the levels of Aβ-degrading enzymes and Aβ transport receptors in brain homogenates (Mann–Whitney *U* tests). *n* = 8. *P <0.05, **P <0.01, ***P <0.001. The error bars are the SEMs.

To further explore the impact of cataracts on AD pathogenesis, we stained with a 6E10 antibody to assess total Aβ plaques. Compared with the control group, the cataract group presented a significantly greater area fraction and density of 6E10 staining in both the neocortex and hippocampus (Fig. 3A). In addition, we used thioflavin S staining to detect compact Aβ plaques. The cataract group presented an increased percentage area and density of thioflavin S staining in the neocortex and hippocampus (Fig. 3B). We also determined the number of plaques in the visual cortex separately and consistently observed that the 6E10 and thioflavin S staining in the visual cortex was more pronounced in the cataract group than in the control group (Fig. 3C). We conducted statistical analysis of the visual cortex areas delineated in an anatomical atlas of coronal sections of the brain published in 2001 [15]. Our findings indicate that cataracts exacerbate the Aβ plaque burden in the brain.

To elucidate the potential mechanism underlying the increase in the number of Aβ plaques, we co-stained with the 6E10 antibody and an anti-IBA1 antibody, which allowed us to visualize microglia and Aβ plaques in the brain and observe the phagocytosis of Aβ plaques by microglia. The results revealed that the phagocytosis of Aβ plaques by microglia in the cataract group was significantly reduced compared to that in the control group (Fig. 3D). These findings suggest that cataract increases the number of Aβ plaques in the brain by impairing the phagocytic function of microglia, thereby affecting the clearance of Aβ plaques.

To further explore this phenomenon, we investigated the levels of proteins associated with Aβ production and Aβ transportation. There were no significant differences in the levels of full-length APP (mutant amyloid precursor protein), its metabolic C-terminal fragments (CTF-β and CTF-α), or the proteases involved in APP metabolism (including BACE1, ADAM10, and PS1) between the cataract group and the control group (Fig. 3E, F). Furthermore, we found that the level of RAGE (Receptor for Advanced Glycation Endproducts), which transports Aβ into the brain, was increased in the cataract group (Fig. 3G). In addition, we examined the level of insulin-degrading enzyme (IDE), an enzyme responsible for the digestion of Aβ in the brain, and found that the expression level of IDE was elevated in the cataract group in a compensatory manner (Fig. 3G). Taken together, our findings reveal that cataract exacerbates Aβ deposition in the brain without interfering with APP metabolism.

### Cataract Augments Neuroinflammation and Neurodegeneration in the Brain

**Fig. 4:**
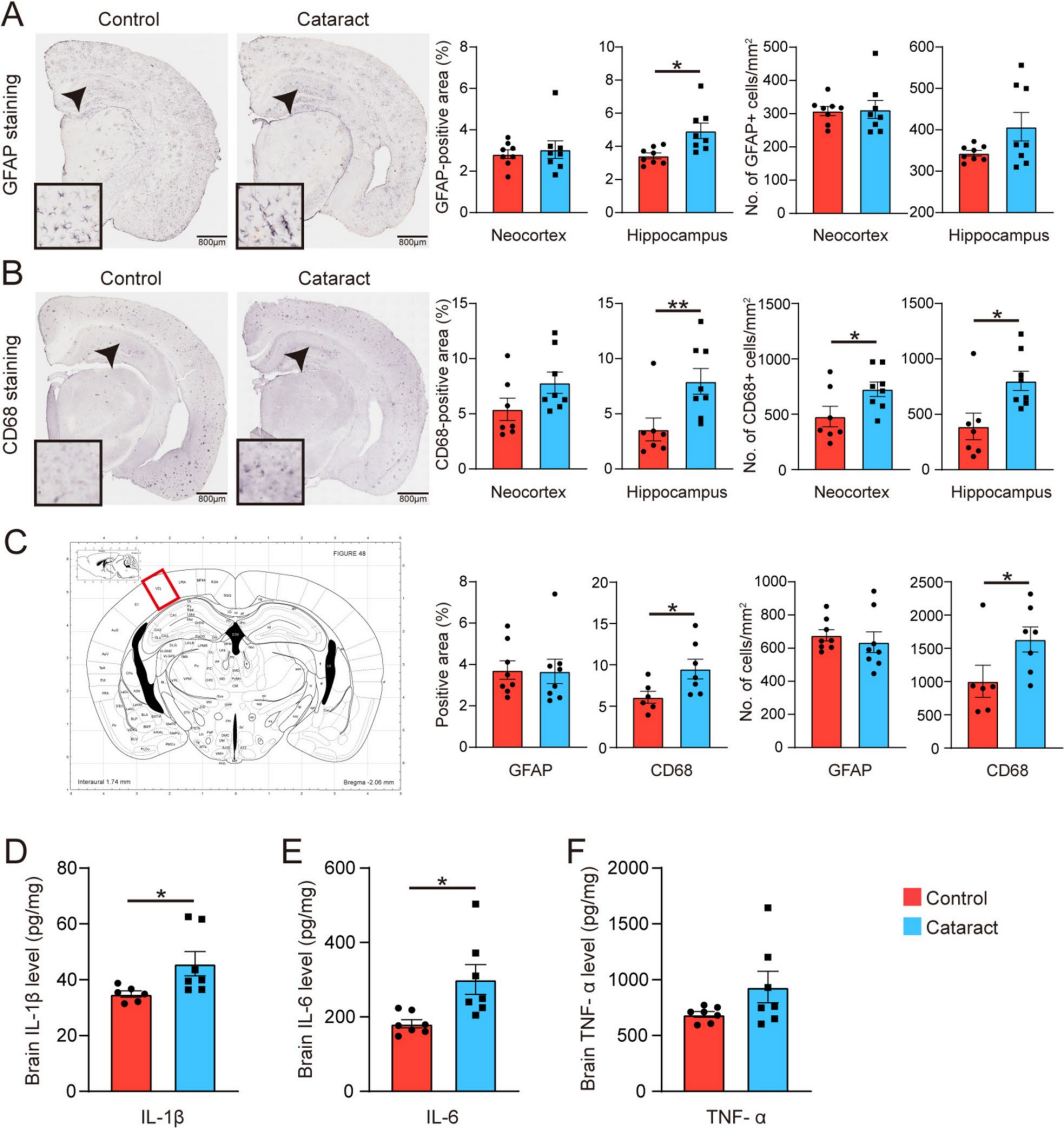
Cataract exacerbates neuroinflammation in AD. **A** Representative images of GFAP staining of astrocytes in brain sections from cataract and control group mice and quantitative results for the neocortex and hippocampus (the statistical charts from left to right represent Mann–Whitney *U* tests, unpaired *t*-tests, unpaired *t*-tests, and Mann–Whitney *U* tests). Scale bars, 200 µm. Insets: magnified views of the areas indicated by the arrows. *n* = 8. **B** Representative images of CD68 staining of microglia in brain sections from cataract and control group mice and quantitative results for the neocortex and hippocampus (the statistical charts from left to right represent three consecutive unpaired *t*-tests followed by one Mann–Whitney *U* tests). Scale bars, 200 µm. Insets: magnified views of the areas indicated by the arrows. *n* = 7–8. **C** Left: image of a coronal brain section; red square indicates the visual cortex. Right: statistical analysis of GFAP and CD68 staining in the visual cortex (the statistical charts from left to right represent Mann–Whitney *U* tests followed by three consecutive unpaired *t*-tests). **D–F** Statistical analysis of proinflammatory cytokine (IL-1β, IL-6, and TNF-α) levels (unpaired *t*-tests). *n* = 6–7. *P <0.05; **P <0.01; ***P <0.001. The error bars are the SEMs.

To explore the impact of cataracts on neuroinflammation in AD model mice, we carried out staining for glial fibrillary acidic protein (GFAP) and CD68 to assess astrogliosis and microgliosis, respectively. Compared with the control group, the cataract group presented a greater number of astrocytes in the hippocampus and an increased number of activated microglia in both the neocortex and hippocampus (Fig. 4A, B). In the visual cortex, there was a significant increase in the number of activated microglia in the cataract group, whereas there was no significant difference in the number of astrocytes (Fig. 4C). In addition, to support the immunohistochemical data, we used ELISA to measure the levels of the proinflammatory cytokines IL-1β, IL-6, and TNF-α. Compared with those in the control group, the levels of proinflammatory factors in the cataract group were significantly greater (Fig. 4D–F). These findings collectively suggest that cataract significantly exacerbates neuroinflammatory responses in the AD model mice.

## Discussion

In this study, we confirmed that cataracts exacerbated cognitive dysfunction and aggravated the deposition of Aβ plaques and neuroinflammatory responses in AD model mice. Our findings provide experimental evidence for the impact of cataracts on the development of AD and emphasize the importance of visual signals in maintaining cognitive well-being.

Early researchers discovered that VI has a significant effect on cognitive function, and subsequent investigations revealed associations between VI and cognitive function in different blinding diseases [16, 17]. Cataracts, the most common cause of blindness, have become a focus of attention. Cataracts were first identified to be associated with AD in 2003 [18]. A retrospective cohort study revealed that, among the comorbidities of AD, the incidence of cataracts is relatively high [19]. Combined with our previous findings, these findings collectively suggest a strong correlation between cataracts and AD, but the cause‒and‒effect relationship between them remains unclear. In our study, we provided *in vivo* experimental evidence that cataract exacerbates cognitive impairment in AD, complementing previous evidence that was solely based on correlative epidemiological data. In addition, we present pathological evidence demonstrating that cataract intensifies pathological changes in the brain associated with AD, such as the Aβ burden and inflammatory response. These results indicate that cataracts may promote the progression of AD; this is consistent with the finding that vision loss is a risk factor for dementia [20]. However, it remains unknown whether these visual deficits can induce changes in neuroplasticity, neuronal activity, and metabolism in specific brain regions. In addition, epidemiological studies also suggest that the prevalence of Parkinson’s disease is increased among individuals with cataracts. The impact of cataracts on AD and other neurodegenerative diseases requires further intensive investigation.

Vision is closely related to the development and maintenance of cognitive functions in the brain. In recent years, transcranial photobiomodulation has emerged as a safe alternative for improving cognitive function in humans [21]. In addition, phototherapy [22], particularly 40 Hz flickering light stimulation, has been found to improve cognitive functions by modulating γ wave activity in animal models, suggesting that visual stimulation is a potential means of maintaining cognitive functions [23]. In addition, a study has proposed that 40 Hz stimulation aids in the clearance of Aβ from the brain, suggesting that such stimulation promotes the microglial phagocytosis of Aβ [24]. Light stimulation at 40 Hz has been proposed as a potential intervention for AD, as it enhances microglial Aβ uptake. Interestingly, our findings also revealed that cataracts hindered Aβ uptake by microglia, suggesting that the increased Aβ load may be associated with reduced light input signals. As highlighted in previous studies, 40 Hz light stimulation modulates γ wave activity and activates neural circuits in key brain regions, which may contribute to cognitive improvement. Furthermore, light stimulation has been shown to restore cortisol and serotonin levels [25], indicating its potential role in regulating neurochemical pathways. Therefore, we hypothesize that cataracts may exacerbate AD pathologies by reducing light input signals, which could indirectly disrupt these neurochemical and hormonal balances. In future studies, we aim to explore this potential mechanism in greater detail and investigate whether enhancing light input signals can mitigate the adverse effects of cataracts on AD progression. Notably, we also found increased levels of RAGE in the brain in the cataract group, which may also be a potential reason for the elevation of Aβ levels in the cataract model, as RAGE can transport Aβ from the blood into the brain parenchyma.

Both AD and cataracts account for a substantial number of clinical cases and thus represent critical public health concerns. Notably, the prevalence of cataracts among AD patients is remarkably high, highlighting the essential need for vigilant attention to the ocular health of AD patients within clinical contexts. This study offers advances in knowledge related to these clinical matters in AD patients who concurrently suffer from cataracts. Given that cataract substantially contributes to the progression of AD and that cataracts can be managed surgically, with contemporary cataract surgery procedures being relatively uncomplicated and effective in restoring patients’ vision [26], our findings compel us to reevaluate the opportune moment for cataract intervention, as such interventions might not only improve patients’ vision but also slow cognitive deterioration. Furthermore, in addition to vision, other sensory functions that are integral to cognitive development could also play a vital role in maintaining brain health and cognitive function.

There are several limitations in our study. First, to minimize the impact of the modelling process on AD, we used a traumatic cataract model, which cannot fully simulate cataracts in humans. Second, despite the use of behavioural tests that do not rely primarily on vision, these tests may not be able to completely exclude the impact of VI on cognitive function. Third, the exploration of the mechanisms by which cataracts affect the progression of AD in this study was relatively superficial, and further in-depth investigation is needed.

In conclusion, our study offers experimental confirmation that cataract considerably contributes to AD pathology and related cognitive impairment, thereby emphasizing the importance of visual stimulation in maintaining cognitive well-being.

## References

[1] Zhang YY, Jiang DQ, Li SS, Liang PJ, Chen AH. Progress in multisensory integration during self motion processing. Sheng Li Xue Bao 2017, 69: 693–702. 29063117

[2] Shang X, Zhu Z, Wang W, Ha J, He M. The association between vision impairment and incidence of dementia and cognitive impairment: A systematic review and meta-analysis. Ophthalmology 2021, 128: 1135–1149. 33422559 10.1016/j.ophtha.2020.12.029

[3] Tran EM, Stefanick ML, Henderson VW, Rapp SR, Chen JC, Armstrong NM. Association of visual impairment with risk of incident dementia in a women’s health initiative population. JAMA Ophthalmol 2020, 138: 624–633. 32297918 10.1001/jamaophthalmol.2020.0959 PMC7163778

[4] Zheng DD, Swenor BK, Christ SL, West SK, Lam BL, Lee DJ. Longitudinal associations between visual impairment and cognitive functioning: The salisbury eye evaluation study. JAMA Ophthalmol 2018, 136: 989–995. 29955805 10.1001/jamaophthalmol.2018.2493 PMC6142982

[5] Burns A, Iliffe S. Alzheimer’s disease. Bmj 2009, 338: b158. 19196745 10.1136/bmj.b158

[6] Alzheimer’s disease facts and figures. Alzheimers Dement 2023, 19: 1598–1695. 36918389 10.1002/alz.13016

[7] Zhang F, Wang JH, Zhao MS. Dynamic monocyte chemoattractant protein-1 level as predictors of perceived pain during first and second phacoemulsification eye surgeries in patients with bilateral cataract. BMC Ophthalmol 2021, 21: 133. 33711968 10.1186/s12886-021-01880-z PMC7953781

[8] Zheng C, Zeng R, Wu G, Hu Y, Yu H. Beyond vision: A view from eye to Alzheimer’s disease and dementia. J Prev Alzheimers Dis 2024, 11: 469–483. 38374754 10.14283/jpad.2023.118

[9] Li C, Geng Z, Yang B, Xiao H, Wang Z, Ye J. Serum Aβ levels are associated with age-related cataract. Neurotox Res 2021, 39: 369–377. 33400179 10.1007/s12640-020-00325-7

[10] Deng X, Geng Z, Yu J, Dai X, Kuang X, Chen X, *et al*. The association between cataract and cognitive functions in older adults: A longitudinal cohort study. J Alzheimers Dis 2023, 91: 1097–1105. 36565122 10.3233/JAD-220963

[11] Jankowsky JL, Slunt HH, Ratovitski T, Jenkins NA, Copeland NG, Borchelt DR. Co-expression of multiple transgenes in mouse CNS: A comparison of strategies. Biomol Eng 2001, 17: 157–165. 11337275 10.1016/s1389-0344(01)00067-3

[12] Ordoñez-Gutierrez L, Fernandez-Perez I, Herrera JL, Anton M, Benito-Cuesta I, Wandosell F. AβPP/PS1 transgenic mice show sex differences in the cerebellum associated with aging. J Alzheimers Dis 2016, 54: 645–656. 27567877 10.3233/JAD-160572

[13] Wang J, Jin WS, Bu XL, Zeng F, Huang ZL, Li WW, *et al*. Physiological clearance of tau in the periphery and its therapeutic potential for tauopathies. Acta Neuropathol 2018, 136: 525–536. 30074071 10.1007/s00401-018-1891-2

[14] Jin WS, Shen LL, Bu XL, Zhang WW, Chen SH, Huang ZL, *et al*. Peritoneal dialysis reduces amyloid-beta plasma levels in humans and attenuates Alzheimer-associated phenotypes in an APP/PS1 mouse model. Acta Neuropathol 2017, 134: 207–220. 28477083 10.1007/s00401-017-1721-y

[15] Paxinos G, Franklin KBJ (2001) The Mouse Brain in Stereotaxic Coordinates, 2nd edn Academic Press, San Diego.

[16] Lynn SA, Johnston DA, Scott JA, Munday R, Desai RS, Keeling E, *et al*. Oligomeric Aβ_1–42_ induces an AMD-like phenotype and accumulates in lysosomes to impair RPE function. Cells 2021, 10: 413. 33671133 10.3390/cells10020413 PMC7922851

[17] Narsineni L, Rao PPN, Pham AT, Foldvari M. Peptide-modified gemini surfactants as delivery system building blocks with dual functionalities for glaucoma treatment: Gene carriers and amyloid-beta (Aβ) self-aggregation inhibitors. Mol Pharm 2022, 19: 2737–2753. 35802484 10.1021/acs.molpharmaceut.2c00088

[18] Goldstein LE, Muffat JA, Cherny RA, Moir RD, Ericsson MH, Huang X, *et al*. Cytosolic beta-amyloid deposition and supranuclear cataracts in lenses from people with Alzheimer’s disease. Lancet 2003, 361: 1258–1265. 12699953 10.1016/S0140-6736(03)12981-9

[19] Liu X, Guan Z, Liang S, Feng S, Zhou Y. Associations of cataract, cataract surgery with dementia risk: A systematic review and meta-analysis of 448, 140 participants. Eur J Clin Invest 2024, 54: e14113. 37874275 10.1111/eci.14113

[20] Livingston G, Huntley J, Liu KY, Costafreda SG, Selbæk G, Alladi S, *et al*. Dementia prevention, intervention, and care: 2024 report of the Lancet standing Commission. Lancet 2024, 404: 572–628. 39096926 10.1016/S0140-6736(24)01296-0

[21] Zhao C, Li D, Kong Y, Liu H, Hu Y, Niu H, *et al*. Transcranial photobiomodulation enhances visual working memory capacity in humans. Sci Adv 2022, 8: eabq3211. 36459562 10.1126/sciadv.abq3211 PMC10936045

[22] Huang X, Tao Q, Ren C. A comprehensive overview of the neural mechanisms of light therapy. Neurosci Bull 2024, 40: 350–362. 37555919 10.1007/s12264-023-01089-8 PMC10912407

[23] Iaccarino HF, Singer AC, Martorell AJ, Rudenko A, Gao F, Gillingham TZ, *et al*. Gamma frequency entrainment attenuates amyloid load and modifies microglia. Nature 2016, 540: 230–235. 27929004 10.1038/nature20587 PMC5656389

[24] Murdock MH, Yang CY, Sun N, Pao PC, Blanco-Duque C, Kahn MC, *et al*. Multisensory gamma stimulation promotes glymphatic clearance of amyloid. Nature 2024, 627: 149–156. 38418876 10.1038/s41586-024-07132-6 PMC10917684

[25] Abiega O, Beccari S, Diaz-Aparicio I, Nadjar A, Layé S, Leyrolle Q, *et al*. Correction: Neuronal hyperactivity disturbs ATP microgradients, impairs microglial motility, and reduces phagocytic receptor expression triggering apoptosis/microglial phagocytosis uncoupling. PLoS Biol 2016, 14: e1002554. 27649285 10.1371/journal.pbio.1002554 PMC5029941

[26] Harding JJ, van Heyningen R. Epidemiology and risk factors for cataract. Eye (Lond) 1987, 1(Pt 5): 537–541. 3328701 10.1038/eye.1987.82

